# Methicillin-resistant *Staphylococcus aureus* and Vancomycin-resistant Enterococci Co-colonization^1^

**DOI:** 10.3201/eid1110.050508

**Published:** 2005-10

**Authors:** Jon P. Furuno, Eli N. Perencevich, Judith A. Johnson, Marc-Oliver Wright, Jessina C. McGregor, J. Glenn Morris, Sandra M. Strauss, Mary-Claire Roghman, Lucia L. Nemoy, Harold C. Standiford, Joan N. Hebden, Anthony D. Harris

**Affiliations:** * University of Maryland School of Medicine, Baltimore, Maryland, USA; † Veterans' Affairs Maryland Health Care System, Baltimore, Maryland, USA; ‡ University of Maryland Medical Center, Baltimore, Maryland, USA

**Keywords:** Vancomycin-resistant Staphylococcus aureus, Methicillin-resistant Staphylococcus aureus, Vancomycin-resistant enterococci, Intensive care unit, Colonization, Risk factors, Outcomes, Research

## Abstract

We assessed the prevalence, risk factors, and clinical outcomes of patients co-colonized with vancomycin-resistant enterococci (VRE) and methicillin-resistant *Staphylococcus aureus* (MRSA) upon admission to the medical and surgical intensive care units (ICUs) of a tertiary-care facility between January 1, 2002, and December 31, 2003. Co-colonization was defined as a VRE-positive perirectal surveillance culture with an MRSA-positive anterior nares surveillance culture collected concurrently. Among 2,440 patients, 65 (2.7%) were co-colonized. Independent risk factors included age (odds ratio [OR] 1.03, 95% confidence interval [CI] 1.01–1.05), admission to the medical ICU (OR 4.38, 95% CI 2.46–7.81), male sex (OR 1.93, 95% CI 1.14–3.30), and receiving antimicrobial drugs on a previous admission within 1 year (OR 3.06, 95% CI 1.85–5.07). None of the co-colonized patients would have been identified with clinical cultures alone. We report a high prevalence of VRE/MRSA co-colonization upon admission to ICUs at a tertiary-care hospital.

## Abstract 2

High prevalence of co-colonization increases risk for colonization or infection by vancomycin-resistant *Staphylococcus aureus*.

Methicillin-resistant *Staphylococcus aureus* (MRSA) and vancomycin-resistant enterococci (VRE) cause nosocomial infections and are associated with increased rates of illness and death (*1**,**2*). Both organisms are now endemic in many healthcare institutions, particularly in intensive care units (ICUs) (*3*). Vancomycin is commonly used to treat infections caused by MRSA; however, recent emergence of *S. aureus* infections with high-level resistance to vancomycin call into question the future effectiveness of vancomycin for these nosocomial infections (*4*). All known vancomycin-resistant *S. aureus* (VRSA) isolates reported thus far have possessed the *vanA* gene, which confers resistance to vancomycin and is believed to have been acquired when an MRSA isolate conjugated with a co-colonizing VRE isolate (*5**–**10*). Thus, patients simultaneously co-colonized with MRSA and VRE are likely at increased risk for colonization or infection by VRSA.

Patients in the ICU and other critically ill patients are at high risk for co-colonization with MRSA and VRE and, possibly, VRSA, since both organisms are endemic and associated with increased illness severity (*11**,**12*). Despite the high risk, epidemiologic risk factors associated with co-colonization by MRSA and VRE in patients admitted to ICUs have not been described. In addition, previous studies in this population have been limited by the use of clinical cultures as markers for colonization, which underestimate the true proportion of patients colonized with these resistant organisms (*13**–**15*).

To our knowledge, this study is the first to assess independent risk factors and outcomes for patients co-colonized with VRE and MRSA. The aim of this study was to estimate the prevalence, risk factors, and clinical outcomes of patients who are co-colonized by VRE and MRSA upon admission to the medical and surgical ICUs of a tertiary-care facility.

## Methods

### Study Design and Patient Population

This study was approved by the institutional review board of the University of Maryland, Baltimore. This study utilized a prospective cohort of adult patients admitted to the medical ICU (MICU) and surgical ICU (SICU) of the University of Maryland Medical Center (UMMC) between January 1, 2002, and December 31, 2003. UMMC is a tertiary-care facility in Baltimore, Maryland. The MICU is a 10-bed, private room unit providing care to patients who have acute or potentially life-threatening medical conditions including hematologic and other malignancies. The SICU is a 19-bed, private room unit providing care to adult patients with solid organ transplantation and abdominal, genitourinary, orthopedic, and otolaryngologic surgery.

During the study period, routine surveillance cultures of the anterior nares for MRSA and perirectal area for VRE were obtained from patients within 48 hours of admission to both ICUs for infection control purposes. Cultures were obtained from an average of 80% of admitted patients. Patients from whom both cultures were not obtained upon ICU admission or had an ICU length of stay <5 hours were excluded. Patients may have had multiple admissions during the study period, and all eligible admissions were included in this analysis.

### Data Collection and Variables

All data were abstracted from the UMMC central data repository that contains the patients' electronic medical records. The validity of these data was assessed by randomly sampling 10% of the patients' electronic data records and comparing them to the original paper medical records. The positive and negative predictive values of this assessment exceeded 99% for both validity measures, which was similar to values seen in previous studies with this same data source (*16**–**19*).

Co-colonization by VRE and MRSA upon ICU admission was defined as a positive surveillance culture of the perirectal area for VRE and a positive surveillance culture of the anterior nares for MRSA within 48 hours of admission to either the MICU or SICU. All coexisting conditions were defined by using International Classification of Diseases, 9th Revision and the Charlson Comorbidity Index (*20*).

### Laboratory Methods

All media and reagents were from BD Biosciences (San Jose, CA, USA) unless otherwise noted. *S. aureus* was isolated from both anterior nares and perirectal cultures. Nares swabs were plated on tryptic soy agar with 5% sheep blood to isolate *S. aureus*. Plates were examined at 24 and 48 hours for creamy, golden β-hemolytic colonies typical of *S. aureus*. Perirectal swabs were plated on both tryptic soy agar with 5% sheep blood and phenylethyl alcohol agar (Remel, Lenexa, KS, USA) and cultured in Baird *Staphylococcus* Enrichment broth (Merck, Darmstadt, Germany). Presumptive *S. aureus* colonies were confirmed by positive catalase and Murex Staphaurex (Remel) reactions. MRSA were identified by growth on Mueller Hinton agar with 4% NaCl and 6 μg/mL oxacillin.

Enterococci were isolated by plating perirectal swabs on Columbia Modified CNA agar and examined at 24 and 48 hours. Presumptive enterococci colonies that were gram-positive cocci, catalase negative, and pyrrolidonyl-β-naphthylamide positive were plated on vancomycin screening agar and motility agar. Vancomycin-resistant, nonmotile enterococci were identified as VRE.

### Statistical Analyses

Three risk factor analyses were conducted: 1) VRE/MRSA co-colonized patients were compared to all other ICU patients, 2) patients colonized with VRE alone were compared to patients not colonized with VRE, and 3) patients colonized with MRSA alone were compared to patients not colonized with MRSA. Patients co-colonized with MRSA or VRE were excluded for analyses 2 and 3 above that compared solitary VRE or MRSA colonization to noncolonized patients.

Student *t*, chi-square, Fisher exact, and Wilcoxon rank sum tests were used for descriptive analyses to assess bivariable differences between groups. All variables that were significant (α = 0.1) in the bivariable analyses were included in the initial (full) multivariable logistic regression model. In each of the multivariable analyses performed, variables not significantly associated (α = 0.05) with the outcome (VRE/MRSA co-colonization and VRE or MRSA solitary colonization upon admission to the ICU) were removed from the model. Each of the removed variables was then reinserted into the model to assess if its presence altered the regression coefficient by >20%. If so, this risk factor was included in the final model. The resulting multivariable logistic regression model was considered the final model and was used to calculate odds ratios and 95% confidence intervals for the remaining risk factors.

Differences in patients' clinical outcomes after assessing colonization status were also assessed. These variables included length of stay after the ICU admission culture, subsequent positive clinical cultures for VRE or MRSA, in-hospital death rate, and readmission to the index hospital or transfer to another healthcare facility. Subsequent positive clinical cultures were limited to sterile sites, and thus only blood, cerebrospinal fluid, or urine cultures were considered.

## Results

During the 2-year cohort period (January 1, 2002–December 31, 2003), 3,090 patients were admitted to the MICU or SICU for a >5-hour stay. Of these, 2,440 patients (79.0%) had both anterior nares and perirectal admission cultures collected. Sixty-five patients (2.7%) were co-colonized with VRE and MRSA, 247 patients (10.1%) were colonized with VRE alone, and 175 patients (7.2%) were colonized with MRSA alone. Of the 57 MRSA/VRE co-colonized patients with perirectal samples available for additional analysis, 23 patients (40.4%) were perirectally colonized with MRSA in addition to perirectal VRE and MRSA nasal colonization. Results of the bivariable analyses for VRE/MRSA co-colonization are displayed in Table 1. Because of space constraints, bivariable results for colonization by MRSA or VRE alone are not shown. These results suggest that patients colonized with VRE, MRSA, or both were significantly more likely to have been admitted to the MICU, have had previous hospital admissions, and have antimicrobial exposures within 1 year of current admission (p<0.05 for all). Among co-colonized patients, 58% had been admitted to the index hospital in the previous year, and 51% had received antimicrobial drugs during an admission in the previous year. In addition, ≈48% of MRSA/VRE co-colonized patients had a previous positive culture (clinical or surveillance) for MRSA before the study period, and ≈28% had a previous positive culture for VRE.

**Table 1:** Characteristics of the study population by co-colonization status*

Characteristic	MRSA/VRE co-colonization (n = 65)	No co-colonization (n = 2,375)	p value
Demographic variable			
Mean age, y (SD)	61.1 (15.1)	55.7 (16.0)	<0.01
Male sex, n (%)	43 (66.2)	1,277 (53.8)	0.05
Variables from current admission			
Type of ICU (MICU), n (%)	49 (75.4)	1,014 (42.7)	<0.01
Transfer from another hospital, n (%)	10 (15.4)	711 (29.9)	<0.01
Comorbidities			
HIV/AIDS, n (%)	3 (4.6)	77 (3.2)	0.54
Malignancy, n (%)	6 (9.2)	423 (17.8)	0.07
Cardiac disease, n (%)	20 (30.8)	486 (20.5)	0.04
Diabetes mellitus, n (%)	14 (21.5)	486 (20.5)	0.83
Liver disease, n (%)	2 (3.1)	182 (7.7)	0.17
Renal disease, n (%)	3 (4.6)	74 (3.1)	0.50
Mean Charlson comorbidity score (SD)	2.1 (2.2)	2.3 (2.3)	0.37
Antimicrobial drugs before culture			
Vancomycin, n (%)	14 (21.5)	226 (9.5)	<0.01
Piperacillin-tazobactam, n (%)	17 (26.2)	398 (16.8)	0.05
Imipenem, n (%)	2 (3.1)	72 (3.0)	0.98
Cephalosporins, n (%)	7 (10.8)	426 (17.9)	0.14
Aminoglycosides, n (%)	4 (6.2)	109 (4.6)	0.55
Fluoroquinolones, n (%)	11 (16.9)	223 (9.4)	0.04
Variables from previous admissions			
Previous MRSA colonization/infection	31 (47.7)	145 (6.1)	<0.01
Previous VRE colonization/infection	18 (27.7)	178 (7.5)	<0.01
Hospital admission (<1 y), n (%)	37 (57.9)	751 (31.6)	<0.01
ICU admission (<1 y), n (%)	17 (26.2)	309 (13.0)	<0.01
Antimicrobial drugs during admission (<1 y), n (%)	33 (50.8)	606 (25.5)	<0.01

*MRSA, methicillin-resistant *Staphylococcus aureus*; VRE, vancomycin-resistant enterococci; SD, standard deviation; ICU, intensive care unit; MICU, medical ICU.

Independent risk factors identified with logistic regression for the different colonization states (co-colonization, VRE only, or MRSA only) were markedly different (Table 2). Table 3 displays clinical outcomes of VRE/MRSA co-colonized patients. Approximately 25% percent of co-colonized patients died during the current admission; however, mortality was not significantly higher compared to non–co-colonized patients (p = 0.15). Three percent of co-colonized patients had MRSA-positive clinical cultures, and 3% of co-colonized patients had VRE-positive clinical cultures on the current or subsequent admissions (within 6 months). None of the co-colonized patients had clinical cultures positive for both organisms. Thirty-two percent of VRE/MRSA co-colonized patients were transferred to another hospital upon discharge from the index hospital compared with 15% of non–co-colonized patients (p<0.01).

**Table 2:** Components of final logistic regression models*

Characteristic	MRSA/VRE co-colonization, OR (95% CI)	VRE colonization, OR (95% CI)	MRSA colonization, OR (95% CI)
Age	1.03 (1.01–1.05)		
Male sex	1.93 (1.14–3.3)		
Admission to MICU	4.38 (2.46–7.81)	1.84 (1.38–2.46)	
Antimicrobial drugs during prior admission (<1 y)	3.06 (1.85–5.07)	3.38 (2.54–4.51)	1.66 (1.20–2.30)
Diabetes mellitus		1.39 (1.00–1.83)	1.83 (1.30–2.58)
Liver disease		1.64 (1.04–2.58)	
Renal disease		2.28 (1.26–4.1)	
Vancomycin		1.54 (1.03–2.31)	
Piperacillin-tazobactam		1.77 (1.27–2.47)	
Imipenem		2.47 (1.40–4.36)	
Fluoroquinolones		2.01 (1.37–2.96)	
HIV/AIDS			2.74 (1.46–5.14)

*Only variables significantly associated with the outcome are included in the final models. MRSA, methicillin-resistant *Staphylococcus aureus*; VRE, vancomycin-resistant enterococci; OR, odds ratio; CI, confidence interval; MICU, medical intensive care unit.

**Table 3:** Outcomes of patients co-colonized with VRE and MRSA*

Outcome	MRSA/VRE co-colonization, n = 65	No co-colonization, n = 2,375	p value
Death, n (%)	16 (24.6)	420 (17.7)	0.15
Mean length (d) of hospital stay (SD)	12.0 (16.5)	14.0 (17.8)	0.37
Subsequent MRSA-positive clinical culture, n (%)	2 (3.1)	34 (1.4)	<0.01
Subsequent VRE-positive clinical culture, n (%)	2 (3.1)	39 (1.6)	0.38
Discharge location			
Home or self-care, n (%)	27 (41.5)	1,467 (61.8)	<0.01
Hospital, n (%)	21 (32.3)	368 (15.5)	<0.01
Rehabilitation facility, n (%)	1 (1.5)	62 (2.6)	0.59
Unknown, n (%)	0	18 (0.8)	0.48

*VRE, vancomycin-resistant enterococci; MRSA, methicillin-resistant *Staphylococcus aureus*; SD, standard deviation.

## Discussion

Increasing VRSA prevalence in the healthcare setting could result in considerable illness and death (*21*). To our knowledge, we present the first estimates of the prevalence, risk factors, and clinical outcomes of patients with VRE and MRSA co-colonization. As has been previously reported, co-colonization with VRE and MRSA has always preceded VRSA colonization or infection, and patients admitted to ICUs are likely a high-risk population (*11**–**13**,**15*). We show that among patients who had admission cultures taken, 4.6% admitted to the MICU and 1.2% admitted to the SICU of a tertiary-care facility during a 2-year period were co-colonized with MRSA and VRE, with an overall co-colonization proportion of 3% in both ICUs together. In addition, the observation that ≈40% of MRSA/VRE co-colonized patients were perirectally colonized with both organisms suggests the potential risk for the exchange of genetic material, which could result in the emergence of VRSA.

None of the 65 VRE/MRSA–co-colonized patients had positive clinical cultures for both VRE and MRSA on current or subsequent admissions to the index hospital. Thus, none would have been identified as co-colonized without the active surveillance culturing program in place at our institution. As has been suggested by previously reported cases of VRSA, early identification of these patients is recommended to minimize antimicrobial selective pressure and enhance infection control efforts to reduce the potential for patient-to-patient transmission (*5**,**7*).

In this study, <25% of co-colonized patients died, and nearly 35% were discharged to other hospitals or rehabilitation facilities, where the risk of transmitting these organisms to other patients is substantial. Considering the potential for colonization, infection, and transmission of VRSA, treating physicians, hospital staff, and infection control personnel at receiving institutions must be adequately prepared to isolate and treat these patients (*22**,**23*). Previous studies of transmission from vancomycin-intermediate *S. aureus* (VISA)- or VRSA-colonized or infected patients show that these organisms can be transmitted to close contacts or other hospitalized patients (*7**,**21**,**24*).

Previous studies have also shown that carriage of VRE or MRSA may be persistent, which would increase the potential for co-colonized patients to transmit either or both of these organisms to other patients (*25**,**26*). A mathematical model of the transmission dynamics of VRE has suggested that persistent colonization is the most important factor for increasing the endemic prevalence of this organism in the hospital (*26*). Furthermore, the potential for prolonged co-colonization could increase the likelihood that these patients would experience sufficient selective pressure for emergence of VRSA.

Recent reviews by Reuf, Fridkin, and Cosgrove et al. have summarized risk factors associated with clinical culture positivity for VRSA and VISA (*4**,**22**,**23*). However, with only 3 reported cases of VRSA and ≈20 reported cases of VISA, the precision of statistical associations has been limited. Recent exposure to vancomycin and recent isolation of MRSA were identified as risk factors for VISA in addition to concurrent colonization or infection with VRE and MRSA. Among co-colonized patients in this study, ≈21% received vancomycin, 26% received piperacillin-tazobactam, and 17% received a fluoroquinolone before culture results.

This study is the first to report the prevalence, risk factors, and clinical outcomes of patients with VRE/MRSA co-colonization upon admission to the ICU. We report several risk-factors for co-colonization, including that the odds of co-colonization for patients admitted to the MICU were >4 times greater than those of patients admitted to the SICU and that patients who received antimicrobial drugs within 1 year of admission had 3 times the odds of co-colonization as patients who had not received antimicrobial drugs during a previous admission.

Ray et al. assessed the prevalence of gastrointestinal *S. aureus* colonization among a convenience sample of 37 patients colonized with VRE and reported that 20 (54.1%) of these patients were also colonized with MRSA (*27*). However, comparing these results to those presented here is difficult, given the stark differences between patient groups. The study by Ray et al. included only hospitalized patients from whom a minimum of 3 stool samples (collected weekly) were obtained (i.e., inpatients with extended lengths of stay). We believe that ICU patients co-colonized with VRE and MRSA are at risk of acquiring and transmitting VRSA because they generally are exposed to greater antimicrobial selective pressure, have extended lengths of stay, greater likelihood of indwelling devices, greater severity of illness, and are more likely to have a history of previous hospitalization and related exposures than patients admitted to general medical wards. Despite these differences, perirectal colonization of both organisms was similar between the patients in the study by Ray et al. and this study (54.1% vs. 40.3%, respectively). Other studies have also suggested a prevalence of VRE/MRSA co-colonization or co-infection ranging from 9.5% to 28.6%; however, these studies relied upon clinical cultures to provide these estimates (*14**,**15*).

A limitation of this study is that investigators were unable to determine the species of the VRE isolates. Historically, *Enterococcus faecalis* has been more likely to be associated with conjugation events and subsequent VRSA colonization or infection compared with *E. faecium* (*7**,**28*). However, only 3 VRSA cases are known, and we are not aware of any biologic explanation for why *E. faecium* would be less likely to be involved in transmission of vancomycin resistance to MRSA compared with *E. faecalis*. Still, these data would have been useful and informative. A previous, hospitalwide study at UMMC suggested that among isolated VRE, the prevalence of *E. faecium* and *E. faecalis* were 87%, and 13%, respectively (*29*).

In summary, these data describe a high prevalence of patients co-colonized with VRE and MRSA on admission to an ICU at a tertiary-care hospital, none of whom would have been detected by clinical culture. Risk factors for VRE and MRSA co-colonization are also described. Given that many of these patients were discharged to other institutions, treating physicians and infection control personnel must be cognizant of the risks for VRSA colonization and infection and use appropriate precautions. Appropriate methods to rapidly detect co-colonized patients must be identified to suppress the emergence of VRSA; limit patient-to-patient transmission of MRSA, VRE, and VRSA; and prevent endemic VRSA colonization in healthcare institutions.
